# Supplementary material for: Evaluation of the insulin-dependent and -independent hypoglycemic effects and understanding their breakdown in the progression of obesity using mice
Source: PLoS One. 2025 Dec 23;20(12):e0337739. doi: 10.1371/journal.pone.0337739 (PMC12725660; doi:10.1371/journal.pone.0337739)

S1 Fig. Blood glucose levels decreased regardless of insulin secretion. Related to Fig 1. Time-courses of blood glucose (A) and insulin levels (B) during the IVGTT with and without somatostatin in 12-week-old chow-fed mice. Orange and blue lines indicate the time courses with and without somatostatin, respectively. Results are expressed as mean ± SE (n = 3).

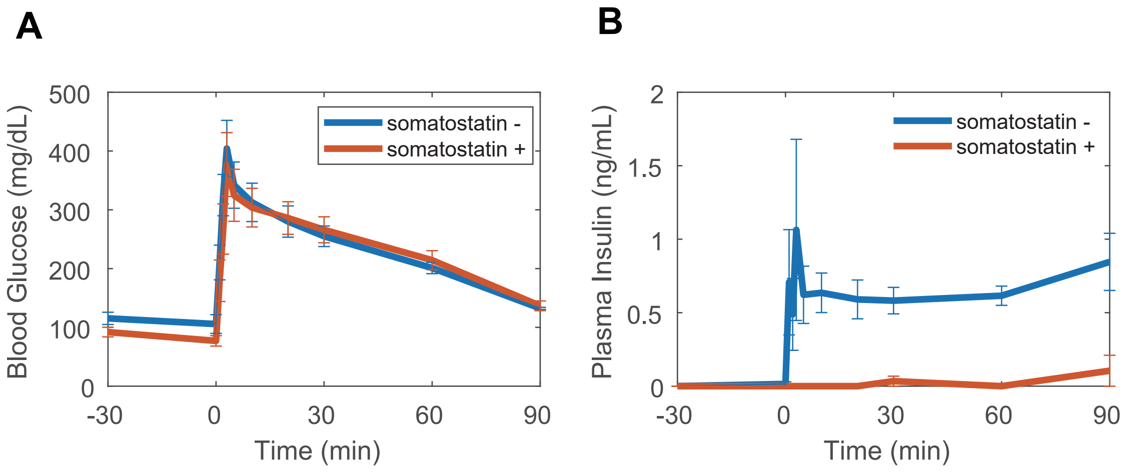

Supplement: S1 Fig — Related to Fig 1. Time-courses of blood glucose (A) and insulin levels (B) during the IVGTT with and without somatostatin in 12-week-old chow-fed mice. Orange and blue lines indicate the time courses with and without somatostatin, respectively. Results are expressed as mean ± SE (n = 3). (PDF) [file pone.0337739.s001.pdf]
