# Supplementary material for: Evaluation of the insulin-dependent and -independent hypoglycemic effects and understanding their breakdown in the progression of obesity using mice
Source: PLoS One. 2025 Dec 23;20(12):e0337739. doi: 10.1371/journal.pone.0337739 (PMC12725660; doi:10.1371/journal.pone.0337739)

S2 Fig. The time courses during the hyperglycemic clamp with 2-DG for the indicated time points. Related to Fig 3.

The time courses of blood glucose (left) and insulin (middle) levels, and the amount of infused glucose (right) during the hyperglycemic clamp with 2-DG for 10, 20, 60, and 120 minutes (n = 3). All results are expressed as mean ± SE. The hyperglycemic clamp test was performed in 14-week-old chow-fed mice with the initial glucose infusion rate of 0.15 μL/g/min and 5 μmol of 2-DG was co-infused from 10 min before each time point. The livers and gastrocnemius muscles were obtained to measure 2-DG uptake at indicated time point.

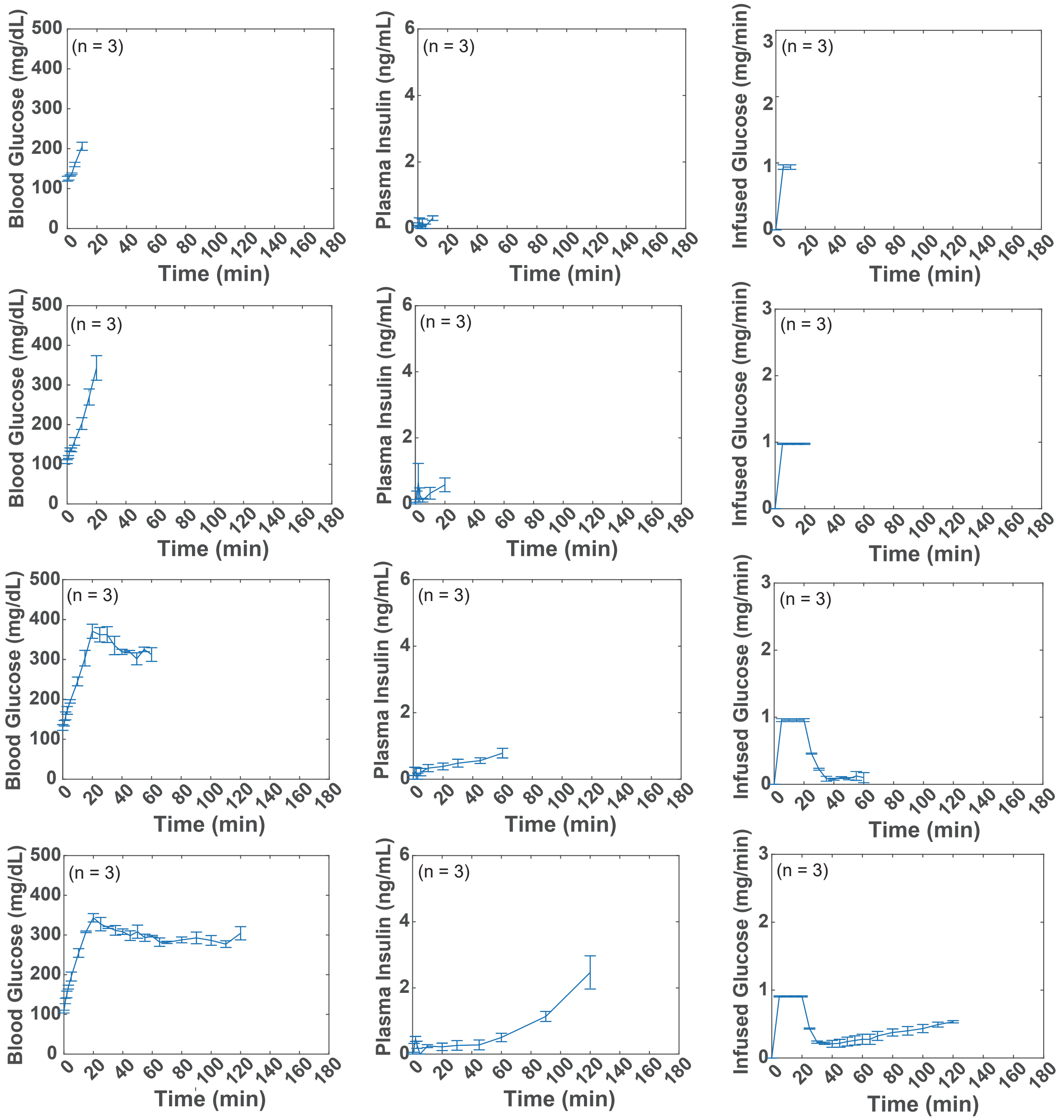

Supplement: S2 Fig — Related to Fig 3. The time courses of blood glucose (left) and insulin (middle) levels, and the amount of infused glucose (right) during the hyperglycemic clamp with 2-DG for 10, 20, 60, and 120 minutes (n = 3). All results are expressed as mean ± SE. The hyperglycemic clamp test was performed in 14-week-old chow-fed mice with the initial glucose infusion rate of 0.15 µL/g/min and 5 µmol of 2-DG was co-infused from 10 min before each time point. The livers and gastrocnemius muscles were obtained to measure 2-DG uptake at indicated time point. (PDF) [file pone.0337739.s002.pdf]
