# Supplementary material for: Evaluation of the insulin-dependent and -independent hypoglycemic effects and understanding their breakdown in the progression of obesity using mice
Source: PLoS One. 2025 Dec 23;20(12):e0337739. doi: 10.1371/journal.pone.0337739 (PMC12725660; doi:10.1371/journal.pone.0337739)
Supplement: S3 Fig — Related to Fig 6. The time courses of blood glucose (left) and insulin (middle) levels, and the amount of infused glucose (right) during the hyperglycemic clamp for each mouse used in the developed models. Orange dots and blue lines indicate experimental and simulation results, respectively. # shows the number of individual mice. (PDF) [file pone.0337739.s003.pdf]

S3 Fig. Experimental results versus mathematical model simulations in 14-week-old chow-fed mice and 10, 14, 18, 28, and 36-week-old HFD-fed mice. Related to Fig 6.
The time courses of blood glucose (left) and insulin (middle) levels, and the amount of infused glucose (right) during the hyperglycemic clamp for each mouse used in the developed models. Orange dots and blue lines indicate experimental and simulation results, respectively.
shows the number of individual mice.

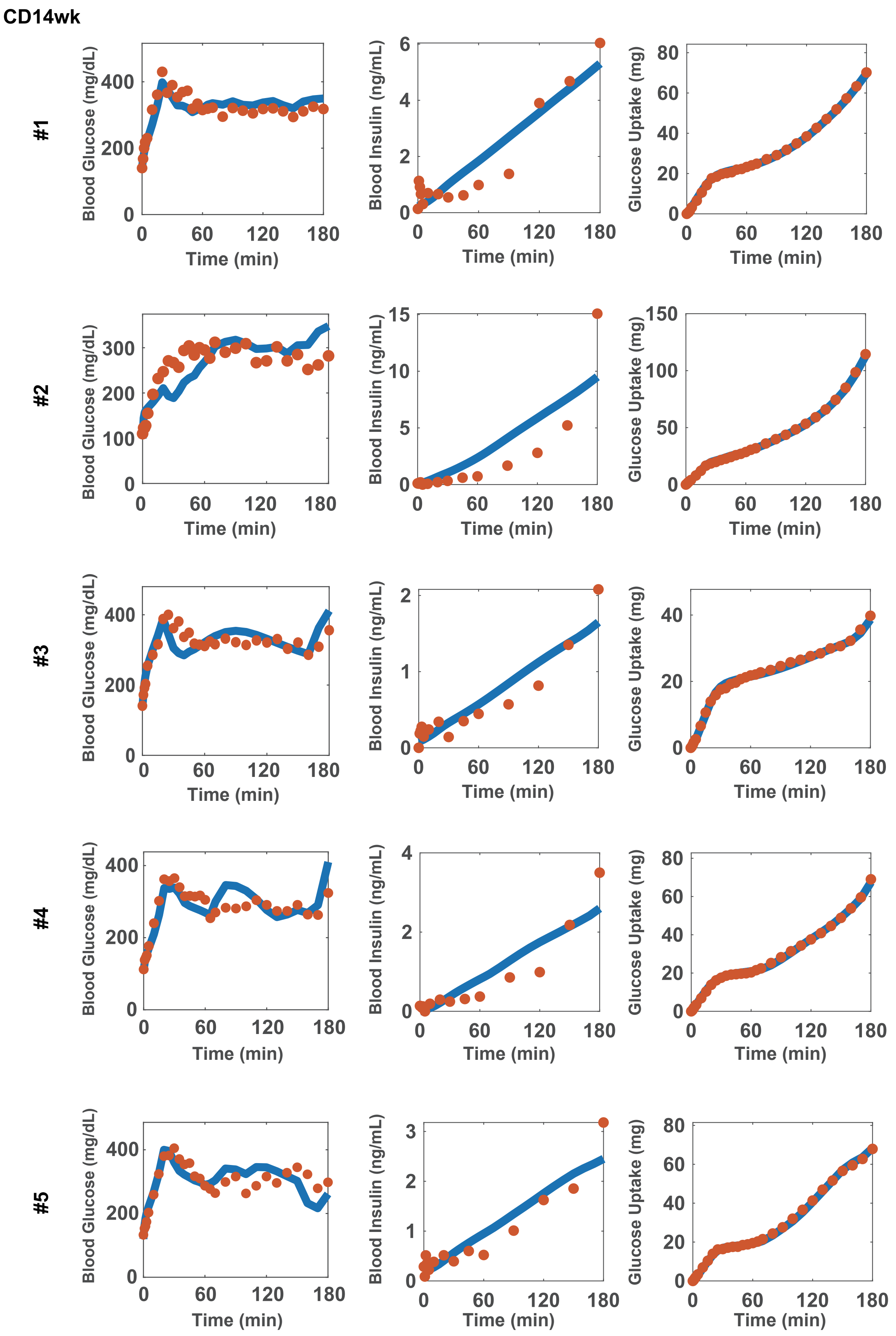

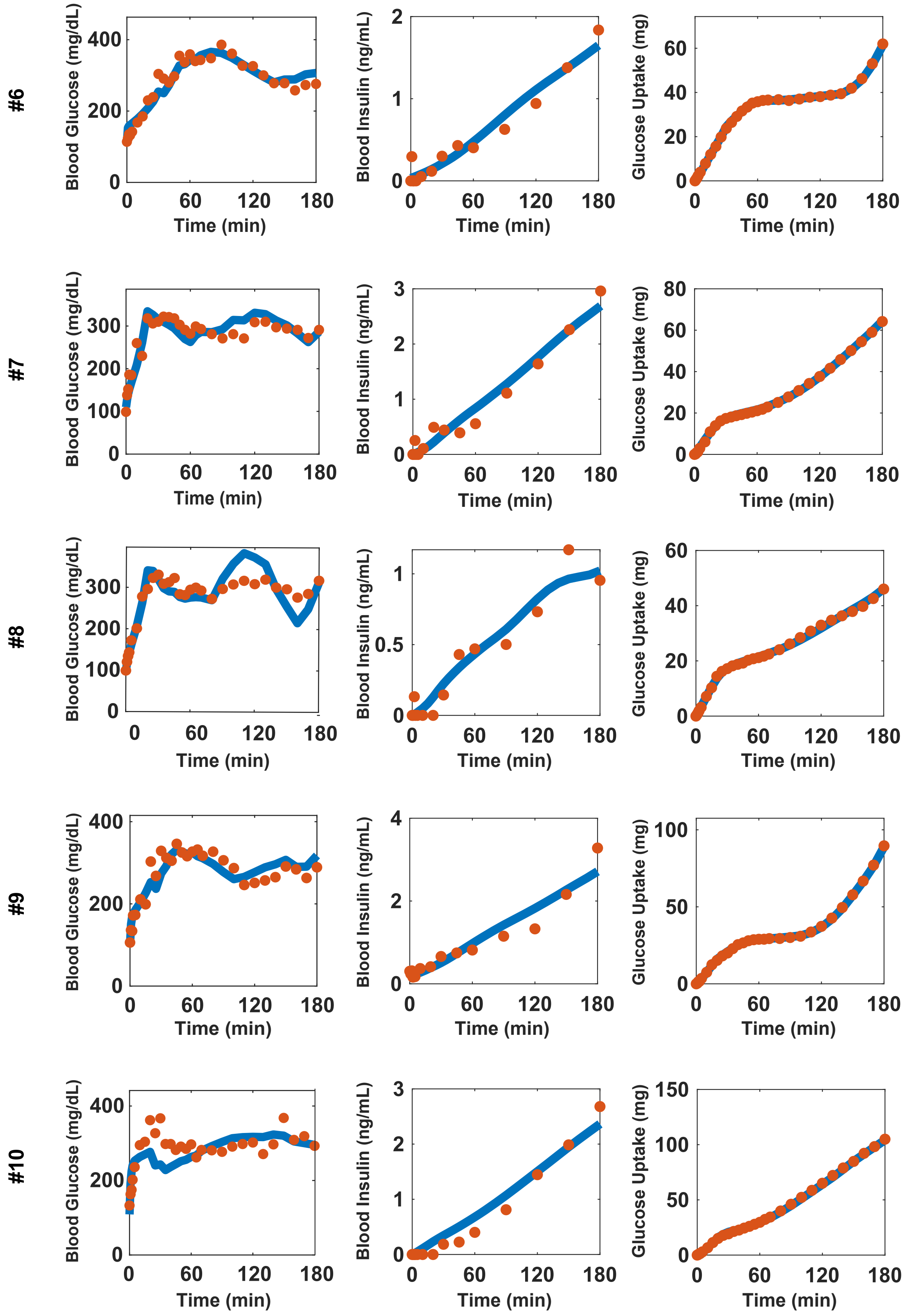

#6
#7
#8
#9
#10
Blood Glucose (mg/dL)
Blood Insulin (ng/mL)
Glucose Uptake (mg)
Time (min)
0
60
120
180

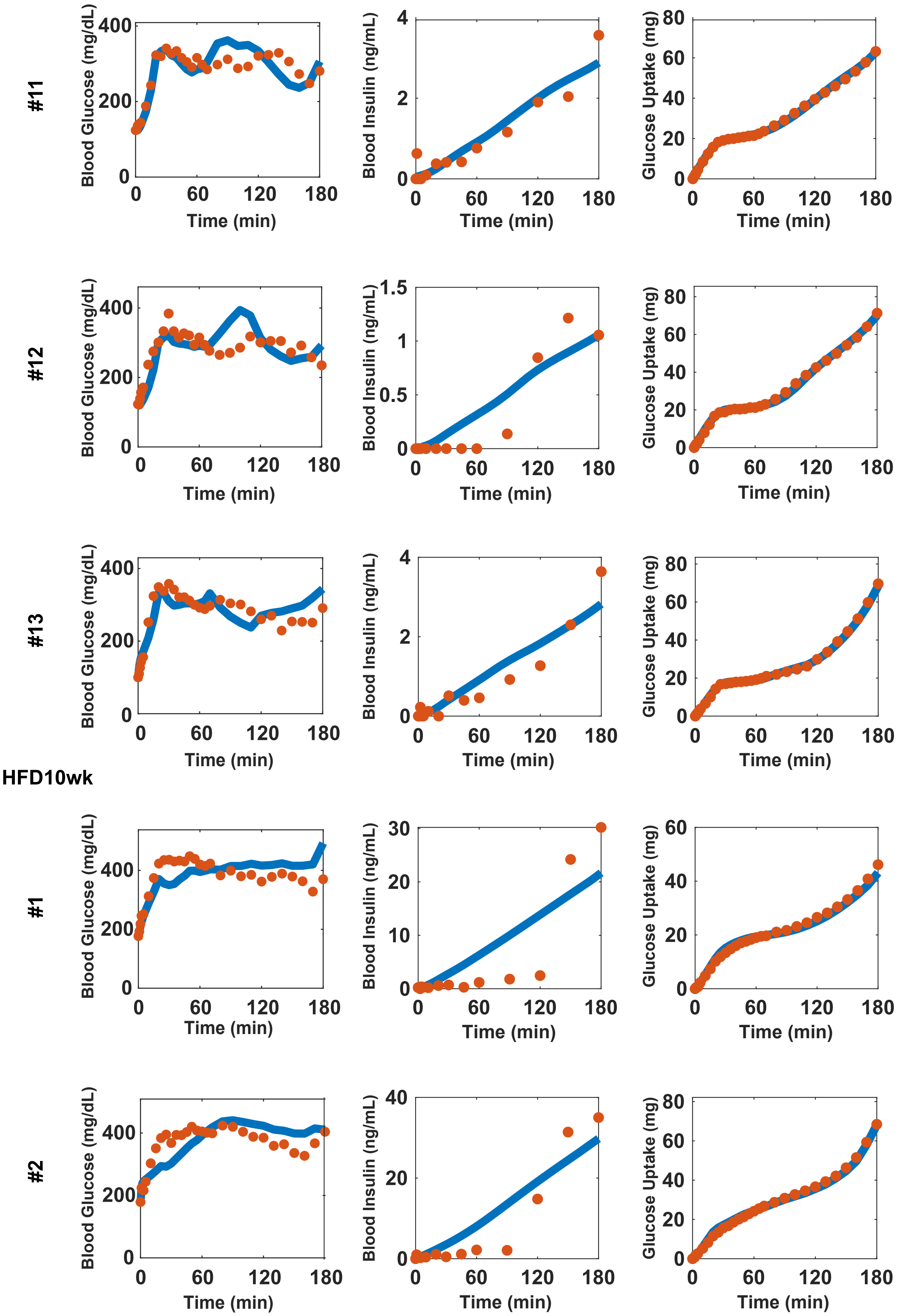
#11
#12
#13
HFD10wk
#1
#2
Blood Glucose (mg/dL)
Blood Insulin (ng/mL)
Glucose Uptake (mg)
Time (min)
0
60
120
180
200
400
0.5
1
1.5
2
4
10
20
30
40
60
80

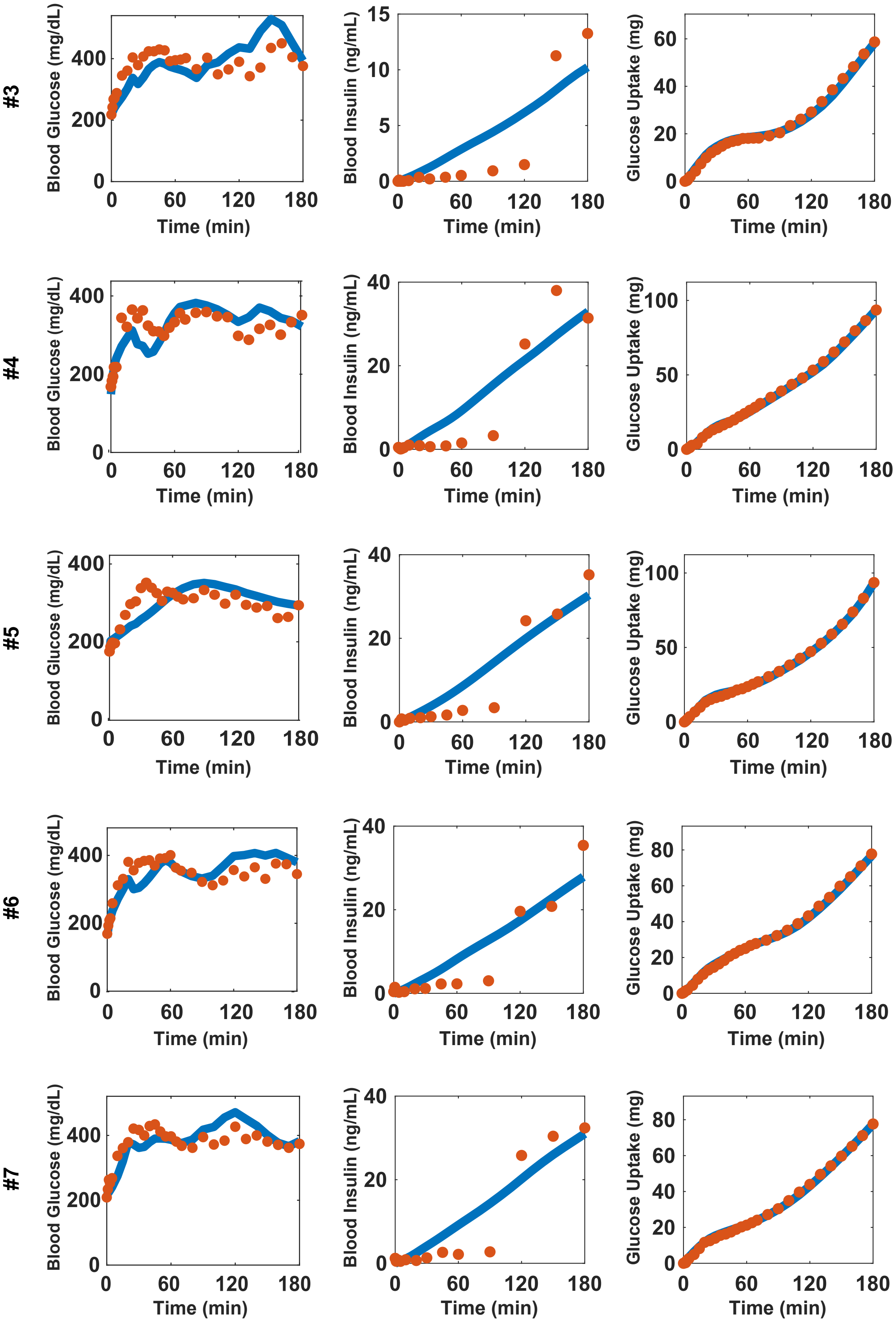
#3
#4
#5
#6
#7
Blood Glucose (mg/dL)
Blood Insulin (ng/mL)
Glucose Uptake (mg)
Time (min)
0
60
120
180
0
200
400
5
10
15
20
40
50
60
80
100

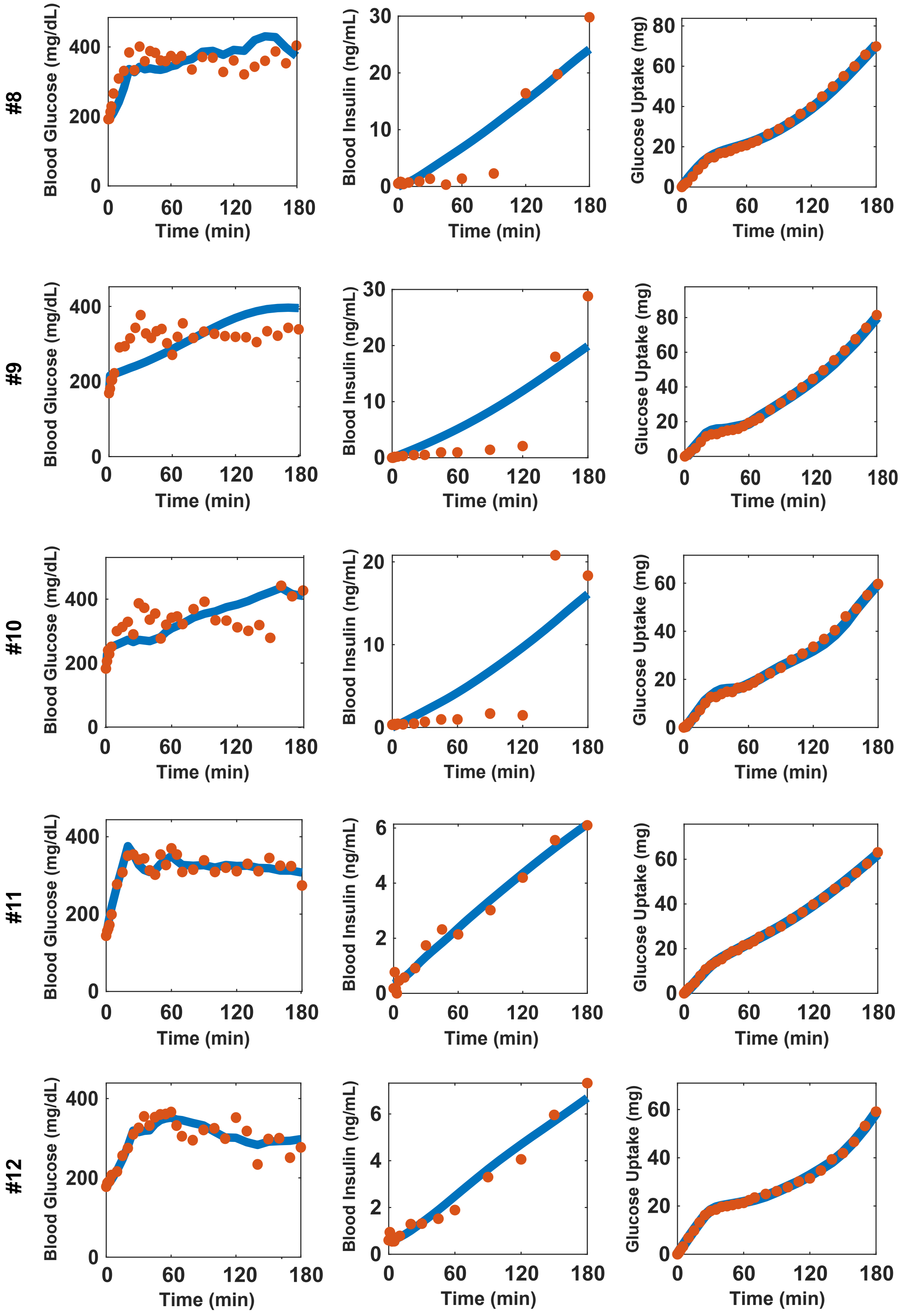
#8
#9
#10
#11
#12
Blood Glucose (mg/dL)
Blood Insulin (ng/mL)
Glucose Uptake (mg)
Time (min)
0
60
120
180

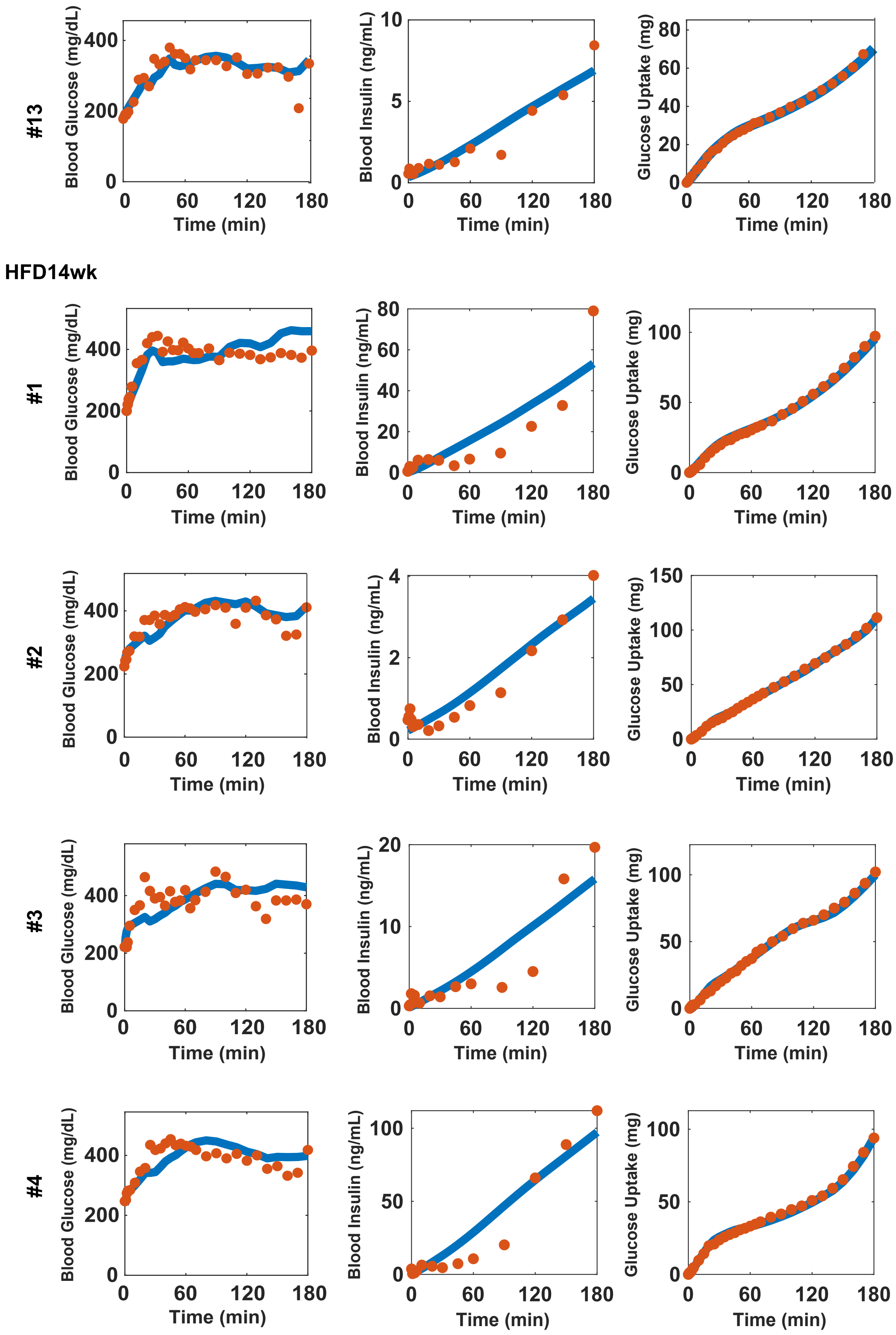
#13
HFD14wk
#1
#2
#3
#4
Blood Glucose (mg/dL)
Blood Insulin (ng/mL)
Glucose Uptake (mg)
Time (min)
0
60
120
180
200
400
5
10
20
40
60
80
50
100
150
2
4

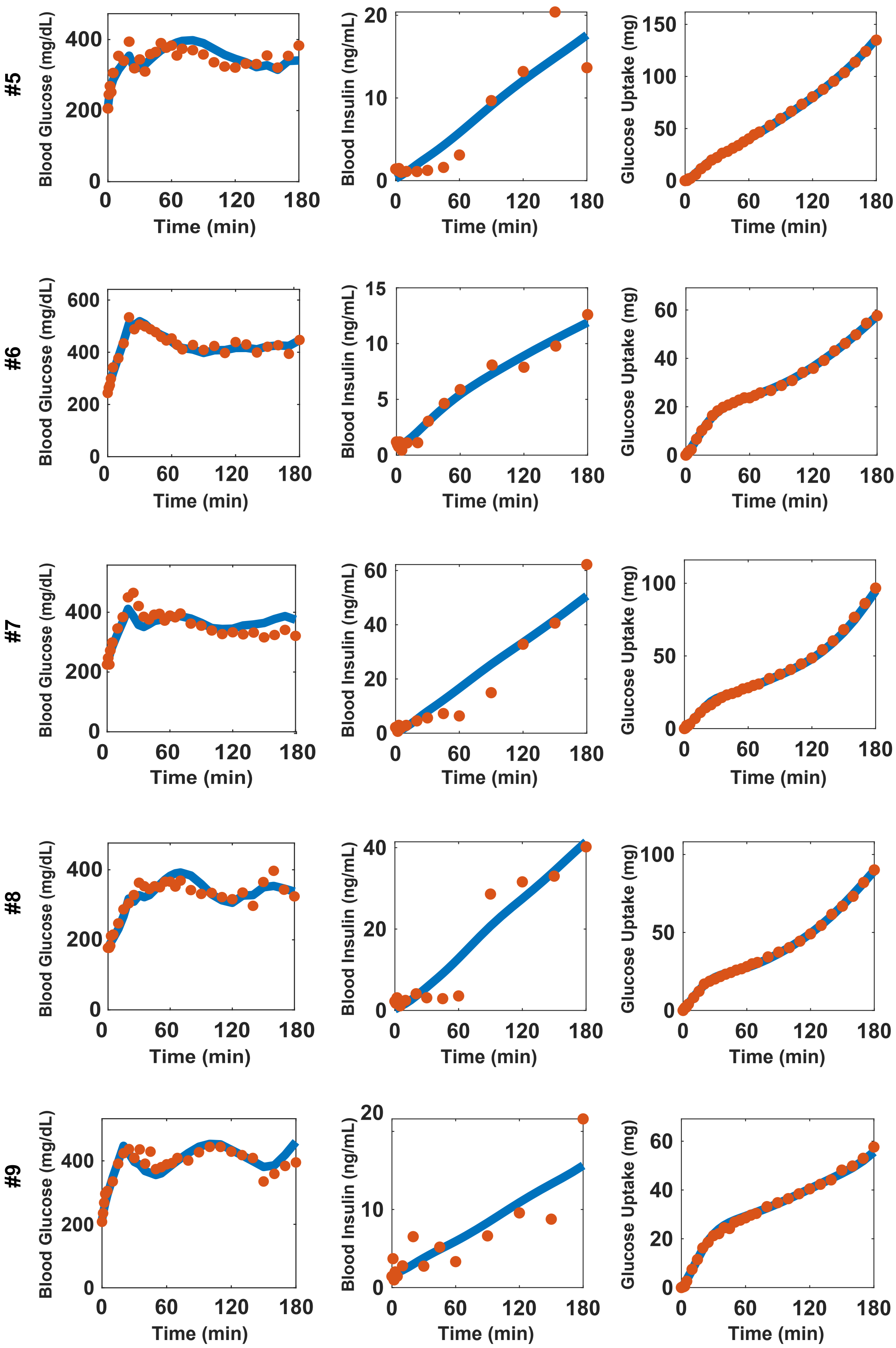

#5
#6
#7
#8
#9
Blood Glucose (mg/dL)
Blood Insulin (ng/mL)
Glucose Uptake (mg)
Time (min)

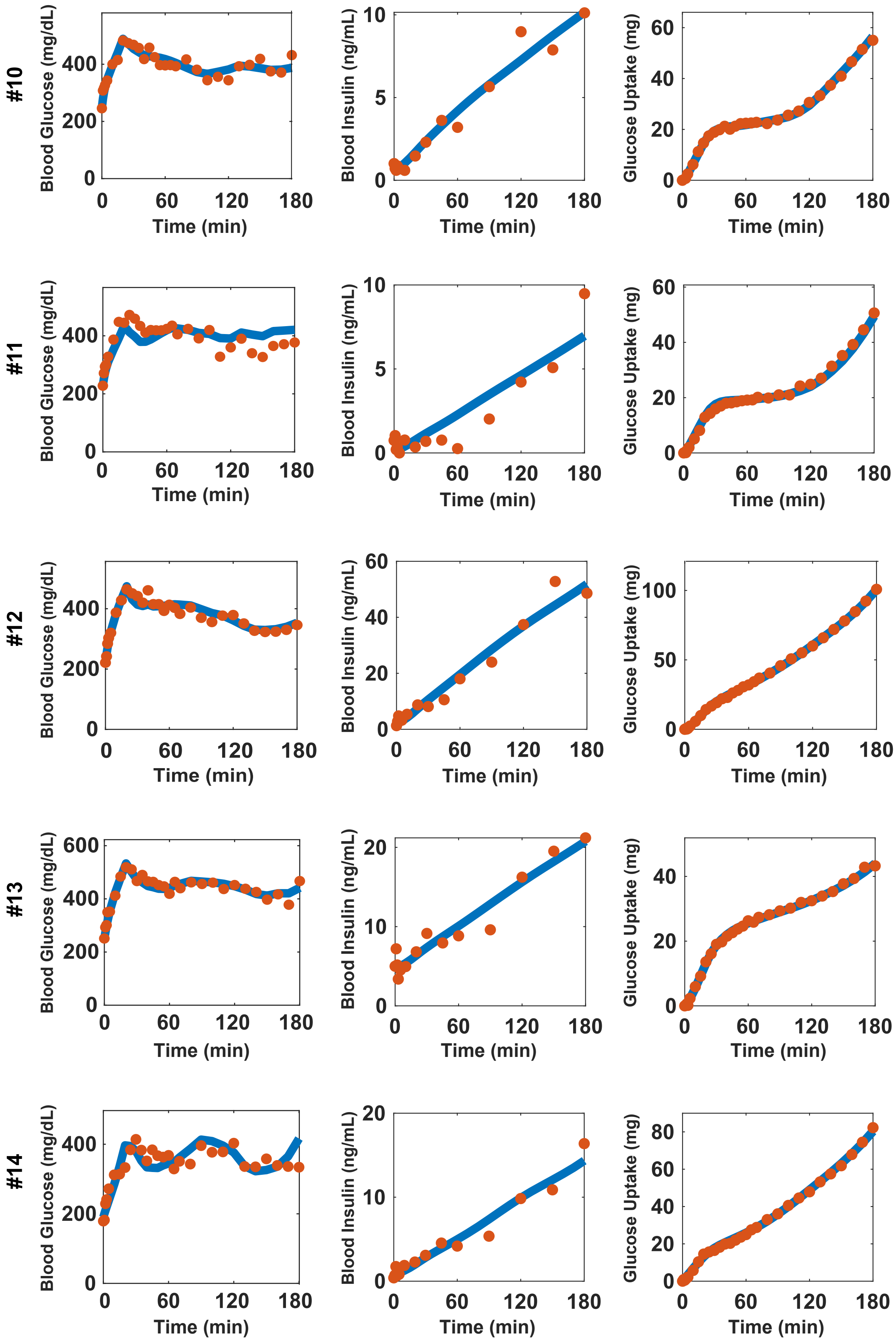
#10
#11
#12
#13
#14
Blood Glucose (mg/dL)
Blood Insulin (ng/mL)
Glucose Uptake (mg)
Time (min)

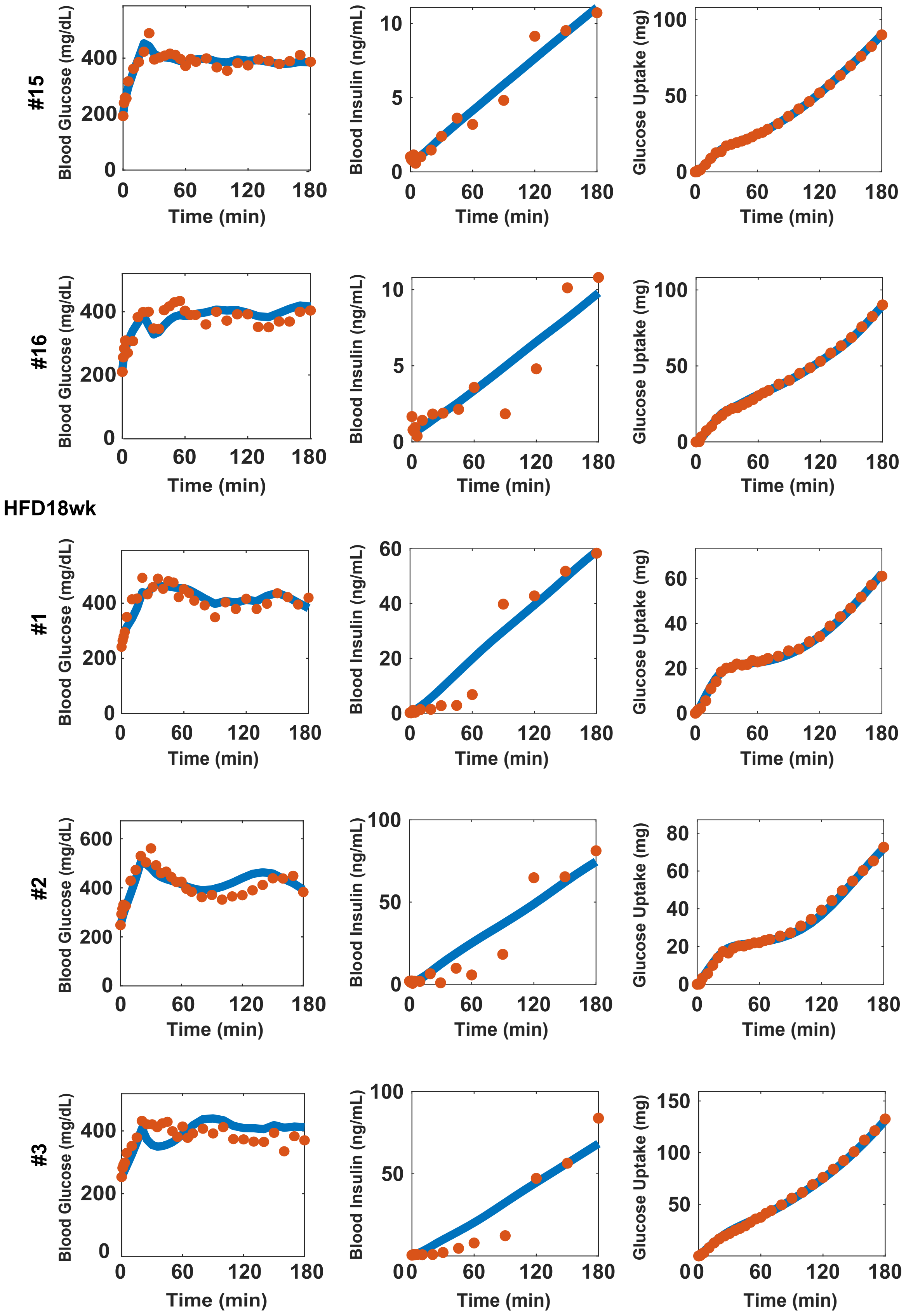

#15
#16
HFD18wk
#1
#2
#3
Blood Glucose (mg/dL)
Blood Insulin (ng/mL)
Glucose Uptake (mg)
Time (min)
0
60
120
180

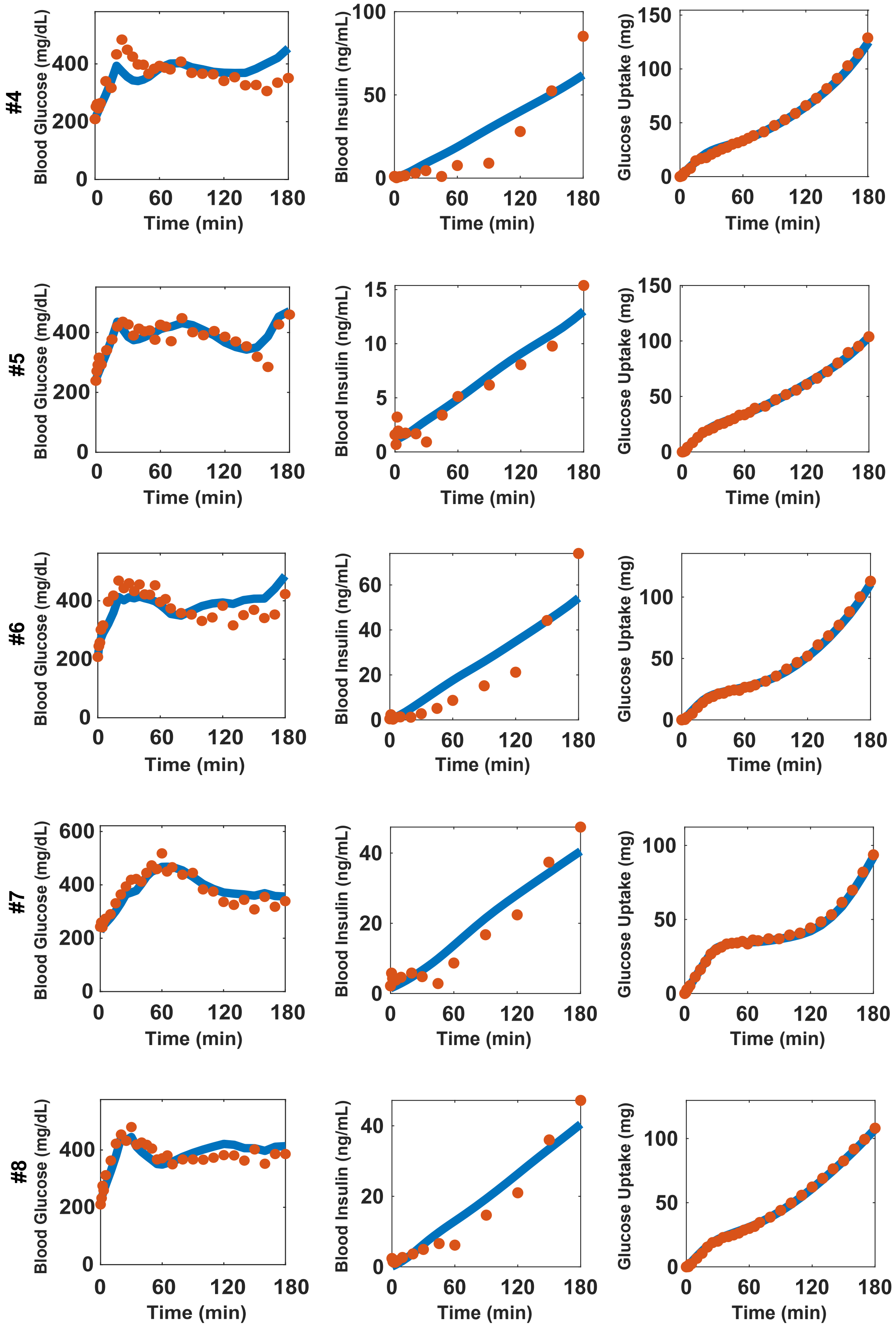
#4
#5
#6
#7
#8
Blood Glucose (mg/dL)
Blood Insulin (ng/mL)
Glucose Uptake (mg)
Time (min)

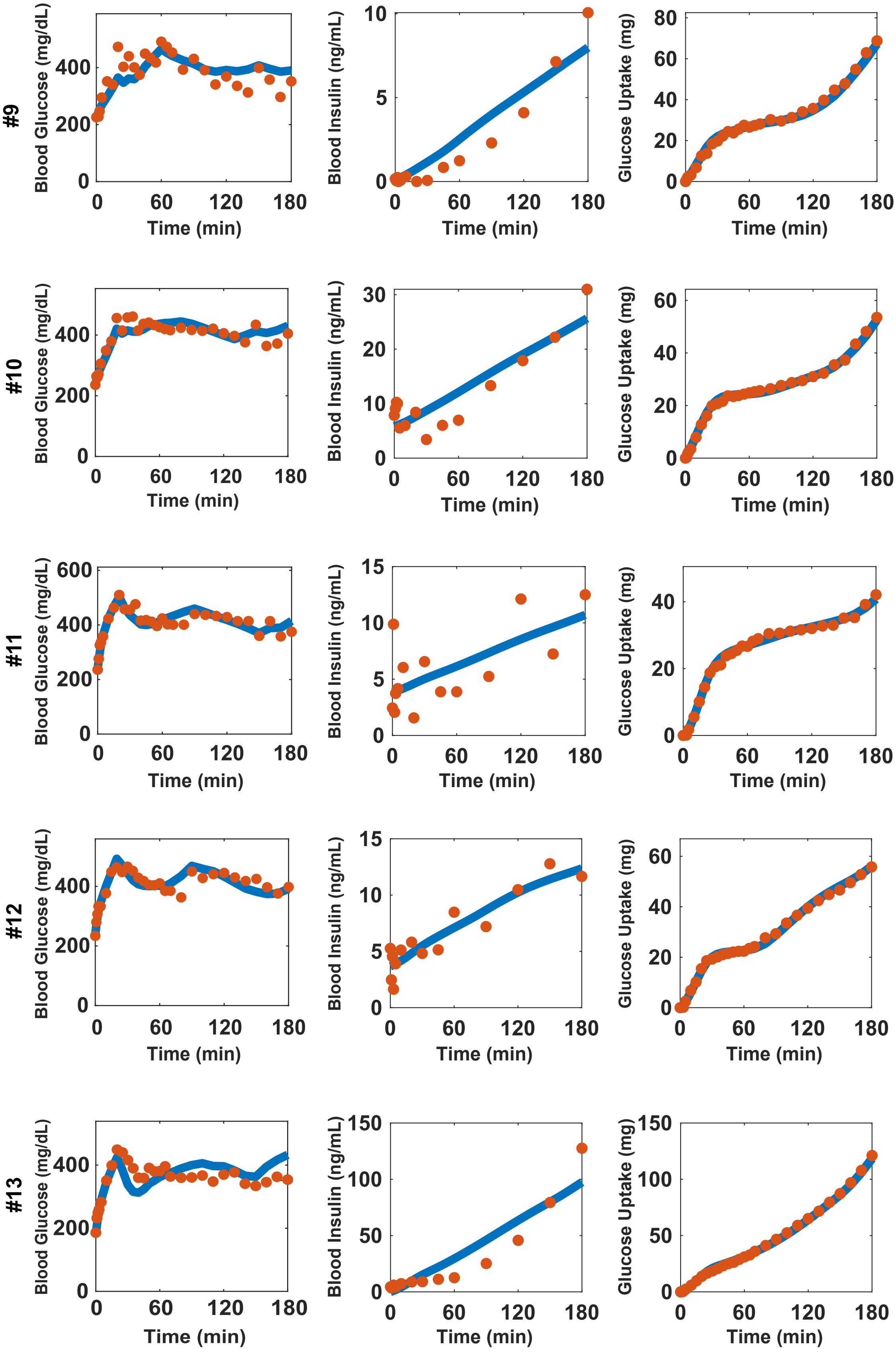

#9
#10
#11
#12
#13
Blood Glucose (mg/dL)
Blood Insulin (ng/mL)
Glucose Uptake (mg)
Time (min)

HFD28wk

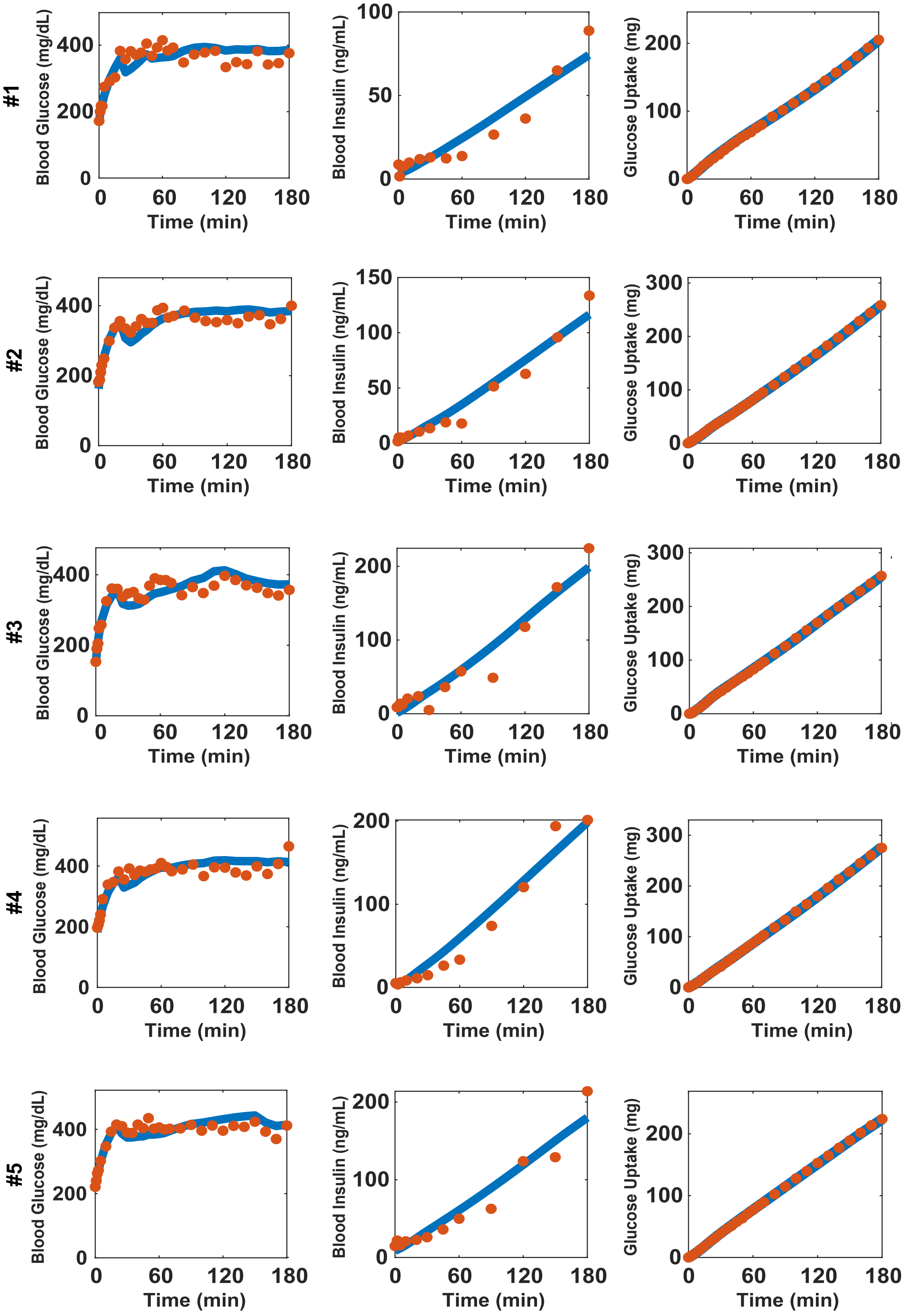

#1
#2
#3
#4
#5
Blood Glucose (mg/dL)
Blood Insulin (ng/mL)
Glucose Uptake (mg)
Time (min)
0
60
120
180

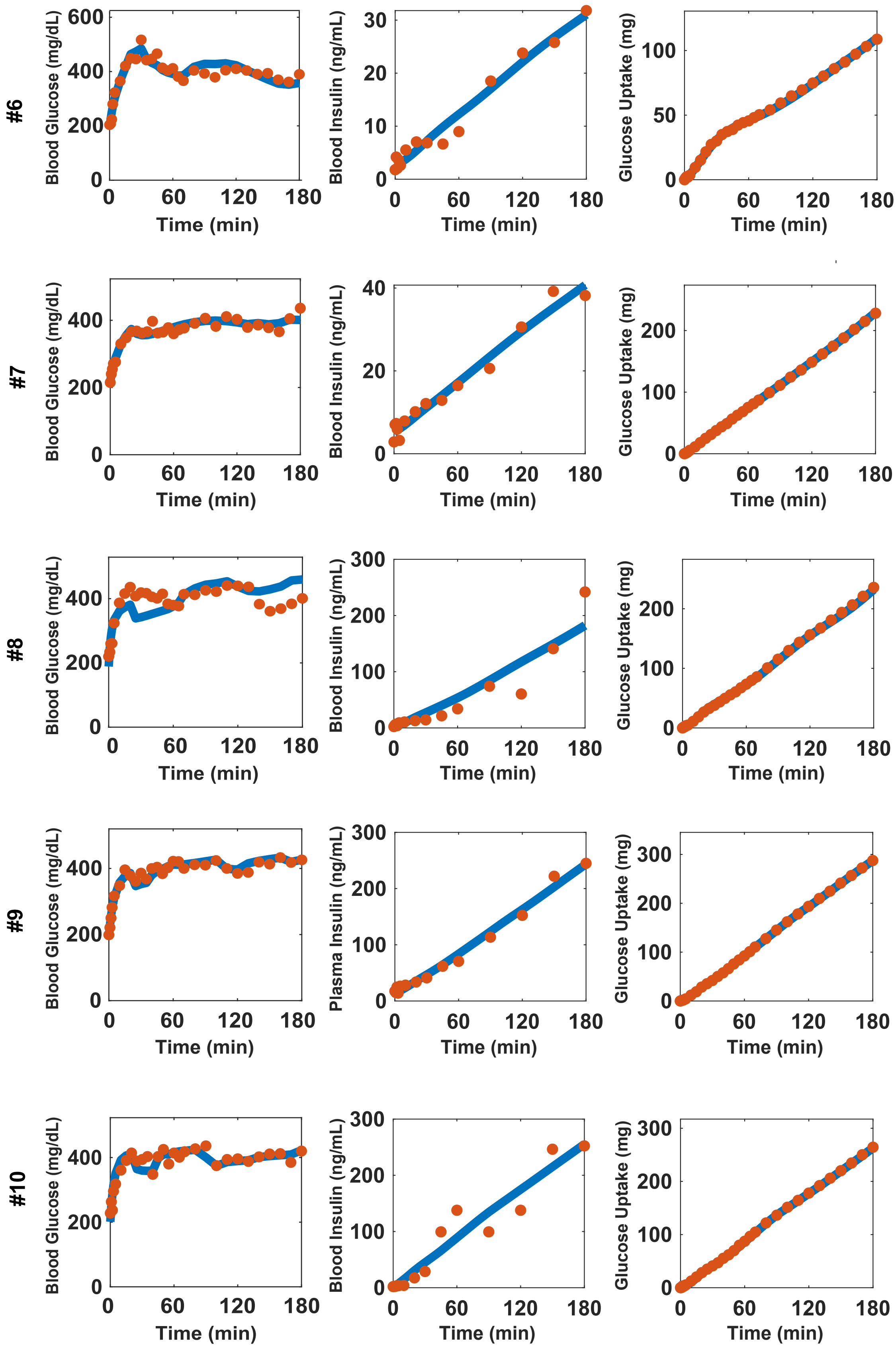

#6
#7
#8
#9
#10
Blood Glucose (mg/dL)
Blood Insulin (ng/mL)
Plasma Insulin (ng/mL)
Glucose Uptake (mg)
Time (min)
0
60
120
180

HFD36wk

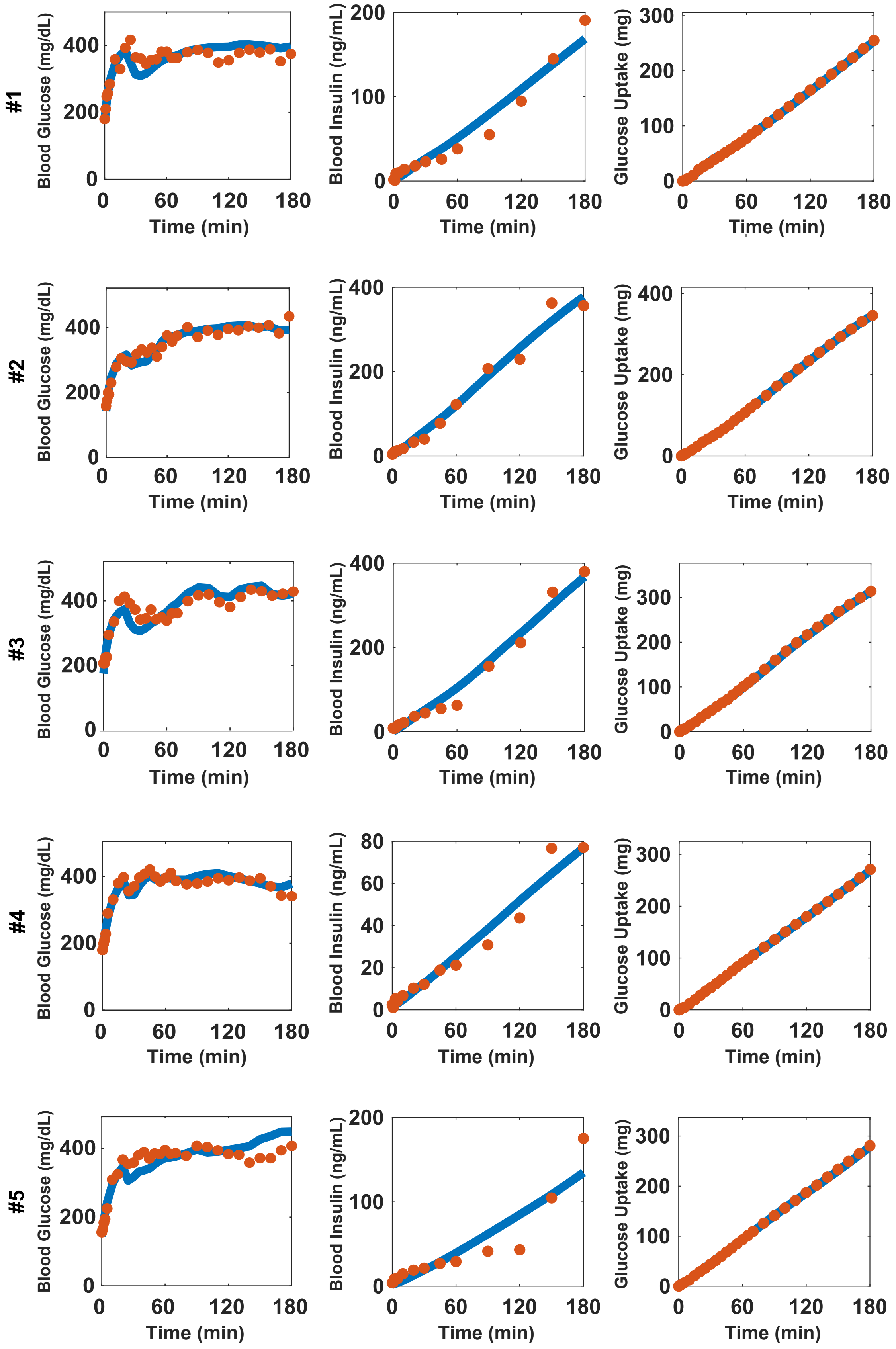

#1
#2
#3
#4
#5
Blood Glucose (mg/dL)
Blood Insulin (ng/mL)
Glucose Uptake (mg)
Time (min)
0
60
120
180

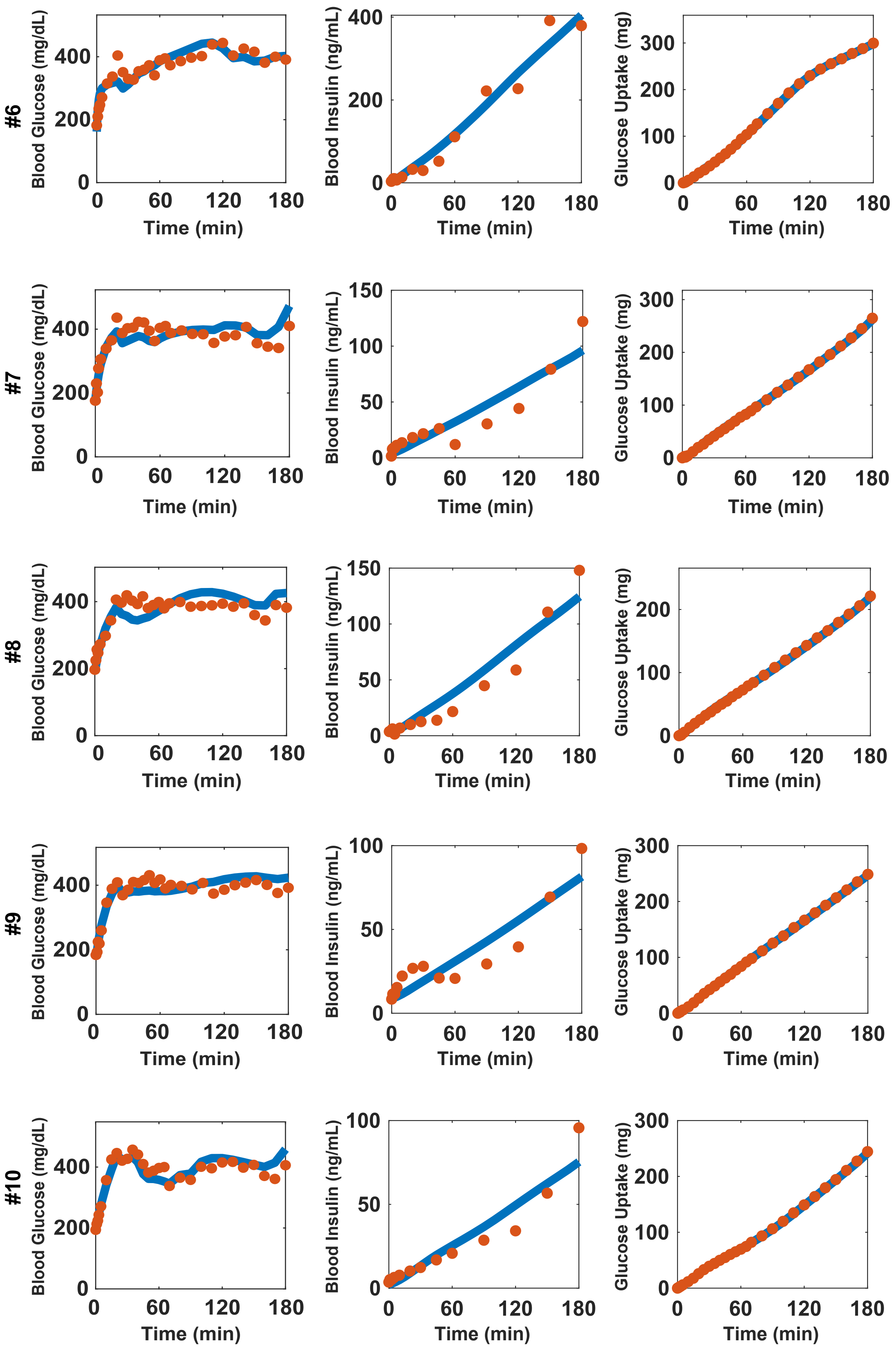
#6
#7
#8
#9
#10
Blood Glucose (mg/dL)
Blood Insulin (ng/mL)
Glucose Uptake (mg)
Time (min)
0
60
120
180
0
200
400
0
50
100
150
0
100
200
300
