# Supplementary material for: Evaluation of the insulin-dependent and -independent hypoglycemic effects and understanding their breakdown in the progression of obesity using mice
Source: PLoS One. 2025 Dec 23;20(12):e0337739. doi: 10.1371/journal.pone.0337739 (PMC12725660; doi:10.1371/journal.pone.0337739)
Supplement: S1 Table — G (0) and I (0) are estimated initial blood glucose and insulin levels. “No” indicates the number of individual mice, which corresponds to the number in Fig 4B, 6, and S3 Fig. (PDF) [file pone.0337739.s004.pdf]

S1 Table. Estimated parameters and RSS for each mouse. *G* (0) and *I* (0) are estimated initial blood glucose and insulin levels. "No" indicates the number of individual mice, which corresponds to the number in Fig 4B, 6, and S3 Fig.

CD 14wk

No	k_1	k_2	k_3	k_4	k_5	k_6	k_7	k_8	*G* (0)	*I* (0)	RSS
1	11291.051	0.0197156	0.0045331	1E-06	0.0110296	0.0013662	27.179278	9996.8937	2.6995706	0.2212	0.075773
2	39956.771	0.0376229	0.0104359	1E-06	0.0080901	1.005E-06	99.371599	9999.3163	1.8183145	1.033E-06	0.2405209
3	233.09079	0.0581526	0.0015231	1E-06	0.0069598	1E-06	37.776944	146.04034	2.3436743	0.0817417	0.0765207
4	1355.8759	0.0593531	0.0028143	1E-06	0.0179385	1E-06	24.390172	1747.4001	1.9186514	1E-06	0.1141312
5	17858.889	0.000001	0.0023176	0.000001	0.0136945	0.0274053	30.771094	8227.2902	2.109607	0.1861422	0.0758545
6	9.2480165	0.2586165	0.001792	1.011E-06	0.008507	0.0008708	186.88968	0.6693921	1.8987329	0.0327159	0.0329533
7	245.38382	1E-06	0.0030505	1E-06	0.0168254	0.0156193	22.364247	693.62185	1.8493557	0.0028969	0.0267752
8	1.231E-06	0.000001	0.0018573	0.0061004	0.0166087	0.0168615	19.368299	9753.2724	1.6764314	1E-06	0.0456181
9	4.331035	0.0679935	0.0027328	1E-06	0.0090544	0.0050837	89.681104	0.4750466	1.8150471	0.1863686	0.0438852
10	24625.85	0.1021436	0.0023492	0.000001	0.0022471	0.000001	196.35023	4163.9373	2.023153	0.000001	0.0535666
11	1798.0579	0.0387154	0.0028698	1E-06	0.0241784	1E-06	22.443461	3937.5277	2.3565313	0.0485247	0.0626147
12	10186.5	0.1414149	0.0010394	1E-06	0.022875	1E-06	28.116761	9995.3598	2.3005496	1E-06	0.1528047
13	17575.918	0.0773233	0.003052	1E-06	0.0183815	1.004E-06	30.918816	9985.417	1.6402306	1E-06	0.0901627

HFD 10wk

No	k_1	k_2	k_3	k_4	k_5	k_6	k_7	k_8	*G* (0)	*I* (0)	RSS
1	17531.218	0.0030012	0.0134751	1E-06	0.0055789	1E-06	34.329952	8270.8772	3.8012855	1E-06	0.2608548
2	157631.92	0.0108083	0.0182921	1.001E-06	0.0034213	1.165E-06	227.65835	7665.3817	4.0757962	2.529E-06	0.1390163

3	1.1039359	1.019E-06	0.0073697	1E-06	0.0070504	0.0019967	51.921003	0.0001012	4.1708228	1.002E-06	0.2251041
4	53.35787	0.0024129	0.0254673	1E-06	0.0034102	0.0004893	61.479403	22.021322	3.2242203	1.067E-06	0.1440057
5	38726.606	0.0398073	0.0238933	0.000001	0.004033	0.000001	603.02194	791.53673	4.0006228	0.000001	0.104432
6	17424.417	1E-06	0.0197757	1E-06	0.004177	0.001546	47.537027	8942.2809	3.5009209	1E-06	0.1165827
7	0.1974002	0.0003479	0.0198145	1E-06	0.0086832	0.0014949	20.743757	0.0001032	4.7897545	1.03E-06	0.1092385
8	0.2692228	0.000001	0.0162899	1E-06	0.0096558	0.0016785	23.481064	0.0001	4.3060791	1E-06	0.1194264
9	405688.86	0.1854526	0.0162859	1E-06	0.0021849	1E-06	7422.3274	1228.4932	3.4672078	1.005E-06	0.2573043
10	114266.52	0.0140683	0.0140701	1.008E-06	0.0024944	1.008E-06	280.55778	9994.7539	3.2194053	7.076E-06	0.2630442
11	2.529E-05	0.000753	0.005138	0.0012014	0.0052795	0.0062164	21.594507	625.85627	3.1622777	0.3477166	0.0129029
12	41660.635	0.0318372	0.0050996	1E-06	0.0092609	1.17E-06	51.565552	8616.5611	3.9624214	0.5085024	0.0204508
13	2.3597378	0.0075827	0.0048815	1E-06	0.005094	0.0012735	45.816451	1.068049	4.0519912	0.351807	0.0584903

HFD 14wk

No	k_1	k_2	k_3	k_4	k_5	k_6	k_7	k_8	G (0)	I (0)	RSS
1	0.2499163	0.0003019	0.0250164	1E-06	0.0043016	0.0005491	32.338462	0.0001001	6.0041629	0.1	0.1716828
2	298.7159	0.0681913	0.0016977	1E-06	0.002367	1.49E-05	179.8691	16.613	6.03303	0.2135116	0.0546079
3	19832.928	0.0064328	0.0077401	0.000001	0.0014329	0.000473	322.35656	843.448	5.9312127	0.1	0.143378
4	176850.87	0.0027412	0.0402674	1E-06	0.0025048	1E-06	200.65725	5610.0294	7.9432823	0.1	0.0745047
5	4.0153703	0.006188	0.0093495	1.048E-06	0.0026679	0.0002685	81.227969	0.3345714	6.1841399	0.2633515	0.082812
6	0.7026943	1E-06	0.0063872	0.0031866	0.0035582	0.0016898	34.181533	0.0001	7.5232574	0.6856105	0.0112654
7	31849.419	0.0025088	0.0251872	0.000001	0.0038395	0.000001	44.620243	6672.4234	6.6557195	0.1	0.0646564
8	1.3505319	0.0004037	0.0230096	1.003E-06	0.0079716	0.000583	37.244283	0.0839342	5.2194774	0.2755914	0.0539148
9	14978.917	3.374E-06	0.0056749	1E-06	0.0024175	0.0011348	46.694637	5829.3968	7.2513044	1.6540201	0.1138592

10	127872.68	0.000001	0.0046827	0.0011382	0.0028427	0.0068168	68.70339	9999.993	7.9432823	0.5394326	0.0202063
11	9.9976083	0.0106746	0.003514	1E-06	0.0036286	0.0007544	53.721506	1.3707348	6.1861773	0.1000001	0.1240064
12	103.50382	0.0017398	0.0231734	1E-06	0.0025105	0.0001451	44.507593	22.717928	6.993836	1.9179594	0.020598
13	4.0543348	1.04E-06	0.0063196	1.001E-06	0.0017863	0.0004933	68.257553	0.0001005	7.9432666	4.6937117	0.0187008
14	1.043E-06	0.000242	0.0078876	1E-06	0.0058804	0.0035355	21.571592	192.65714	5.1155793	0.7537648	0.0304778
15	39567.988	1E-06	0.005508	1E-06	0.004	0.0084201	38.470093	9999.9873	5.3483915	0.6696887	0.0163437
16	71477.527	1.011E-06	0.0045053	1E-06	0.0019222	0.004025	97.091469	9773.4805	6.3419081	0.5590872	0.0782633

HFD 18wk

No	k_1	k_2	k_3	k_4	k_5	k_6	k_7	k_8	G (0)	I (0)	RSS
1	4.6739916	1E-06	0.0248537	1E-06	0.0052875	0.0002777	52.478882	0.145373	7.5194514	0.1	0.0548827
2	56709.969	0.000001	0.0286978	0.000001	0.0036354	0.0007942	39.281396	9995.9112	8.3176377	0.1	0.089051
3	0.000001	0.0012509	0.0298131	1E-06	0.0032591	1E-06	33.266909	1887.5644	8.0982633	0.1	0.0916238
4	20925.021	0.0016083	0.0259192	1E-06	0.0044835	0.000001	34.765913	9997.9301	7.4287921	0.1	0.1523259
5	0.3800531	0.0015458	0.0049629	0.000001	0.0035292	0.0020619	34.923137	0.0001	8.3176377	1.0837206	0.0437305
6	4.9840775	0.0018106	0.0238024	1E-06	0.0057978	1.045E-06	52.408602	0.1679144	6.5914942	0.1	0.1221204
7	136.92232	0.0054365	0.0150831	1E-06	0.0034333	1.808E-06	182.85172	3.1935137	8.3175535	1.4825878	0.0548521
8	0.408148	1E-06	0.0167992	1E-06	0.0044504	0.0014082	30.559706	0.0001	7.3799744	0.1	0.0460262
9	12.010123	0.010626	0.0036417	1.001E-06	0.0037615	0.000618	74.196646	1.1121109	6.6786521	0.1000004	0.0904404
10	113973.21	0.0038306	0.0079557	0.000001	0.0042936	0.000001	52.030986	9988.1297	7.9671302	5.8326353	0.0669263
11	7.5216692	4.868E-05	0.0026559	1.002E-06	0.0011697	0.0009539	155.43757	0.0001053	8.2194901	3.8482105	0.14688
12	119329.74	0.000001	0.0053322	0.0030012	0.0027343	0.0037577	63.383684	9999.9371	7.8598938	3.6805419	0.0315346
13	16913.16	0.0011572	0.0449141	1E-06	0.0022818	1E-06	53.01643	4510.4716	5.8017037	0.1	0.0971003

HFD 28wk

No	k_1	k_2	k_3	k_4	k_5	k_6	k_7	k_8	G (0)	I (0)	RSS
1	0.000001	0.000001	0.0300614	0.000001	0.0011291	0.0005102	122.77303	631.50983	6.1232315	3.1285084	0.0565053
2	0.0016051	0.000001	0.046281	0.000001	0.000453	0.0003726	283.45467	4073.0487	6.2344591	1.5848932	0.028761
3	1832.4387	0.0004544	0.0734296	1E-06	0.0007173	1.002E-06	213.68573	448.97746	6.1615799	1.6459661	0.0429807
4	2.517E-06	0.000001	0.0732944	1E-06	0.0003516	0.0001904	354.92321	9403.7398	7.0141044	1.5848932	0.0305446
5	1E-06	0.0002544	0.065167	1E-06	0.0004594	1E-06	228.41784	139.88027	7.6867794	8.7975563	0.036955
6	34642.68	0.000001	0.0120029	0.000001	0.0022022	0.0011274	67.459494	9474.34	6.3767192	2.5914267	0.0163692
7	1.048E-06	0.0004058	0.0186573	0.0016409	0.0005716	0.0006541	212.34497	143.23902	7.2821727	5.2201541	0.0121329
8	185161.37	0.0003162	0.0636279	1.003E-06	0.0005017	3.09E-05	454.3216	9312.4395	7.3183977	1.5849063	0.101416
9	12496.699	5.029E-05	0.0810806	1E-06	0.0001315	3.82E-06	1119.7577	1772.2473	7.5384514	13.983754	0.0073199
10	31779.365	1.003E-06	0.1004023	0.0012085	5.311E-05	1.452E-06	3177.6432	2055.1809	7.9675092	1.5848946	0.045191

HFD 36wk

No	k_1	k_2	k_3	k_4	k_5	k_6	k_7	k_8	G (0)	I (0)	RSS
1	5417.919	0.0004402	0.0640907	1E-06	0.0004774	9.392E-06	294.50288	1147.9962	6.7095646	1.5135627	0.0310518
2	27608.745	1.026E-06	0.1601408	0.0026041	0.000132	1E-06	1187.2587	9999.9828	6.3423052	1.514489	0.0130351
3	37.138972	0.000001	0.1143335	1E-06	0.0001828	0.000001	776.9435	5.0477078	7.9427693	1.5135613	0.0184496
4	1.093972	0.0005626	0.0268008	1E-06	0.0005034	0.0001171	245.89423	0.2428909	6.9425264	1.7741936	0.0211437
5	0.00389	0.0001674	0.0469036	1E-06	0.0003578	3.776E-05	362.37252	8329.6308	6.0256044	1.9290506	0.094169
6	166329.42	1E-06	0.1437117	1E-06	0.0003494	1.405E-05	978.90063	5470.439	6.6235826	1.5135623	0.0254278
7	22644.561	0.000784	0.0329229	1E-06	0.0006449	6.56E-05	204.26439	6065.648	7.2037387	3.9251265	0.081163

8	1.039E-06	0.0007243	0.0477436	0.000001	0.001172	0.000001	106.37038	9263.4903	6.9366148	1.5135612	0.0538818
9	5.535E-06	0.0004857	0.0257775	1E-06	0.0004119	3.815E-05	255.88145	153.46847	6.9616889	8.7096355	0.0628696
10	1.037E-06	0.000001	0.0258477	0.000001	0.0014629	0.0017381	79.618459	2093.0562	7.2799452	2.0722987	0.0570396
